# Highly efficient organic–graphene hybrid photodetectors *via* molecular peripheral editing†

**DOI:** 10.1039/d4tc02010c

**Published:** 2024-08-12

**Authors:** Shuting Dai, Miao Xie, Can Wang, Yuying Wang, Bin Han, Shunqi Xu, Kexin Wang, Anna Zhuravlova, Bin Xu, Lifeng Chi, Wenjing Tian, Paolo Samorì, Zhaoyang Liu

**Affiliations:** a State Key Laboratory of Supramolecular Structure and Materials, Jilin University Changchun 130012 China zhaoyangliu@jlu.edu.cn; b ISIS, Université de Strasbourg and CNRS 8 allée Gaspard Monge Strasbourg 67000 France samori@unistra.fr; c Institute of Functional Nano & Soft Materials (FUNSOM), Soochow University 199 Renai Road Suzhou 215123 China; d Key Laboratory of Biomass Chemical Engineering of Ministry of Education, College of Chemical and Biological Engineering, Zhejiang University Hangzhou 310027 China

## Abstract

Hybrid systems based on graphene and organic molecules are highly appealing for “correcting” the limited optoelectronic properties of 2D materials. However, an in-depth understanding of the correlation between the structure of the molecular sensitizer and the physical properties of the hybrid toward high-performance organic–graphene hybrid photodetectors remains elusive. Herein, an *ad hoc* molecular design *via* a peripheral editing approach on the organic molecules is employed to elucidate the structure–property relationship when interfaced with graphene forming hybrid systems. Efficient doping of graphene can be attained by physisorption of tetrathiafulvalene molecules exposing electron-donating peripheral groups, benefiting from a strong coupling yielding efficient charge transfer, ultimately leading to photodetectors with an ultra-high responsivity of 1.1 × 10^7^ A W^−1^ and a specific detectivity of 6.5 × 10^14^ Jones, thereby outperforming state-of-the-art graphene-based photodetectors. These results offer valuable insights for future optimization of graphene-based photodetectors through molecular functionalization.

## Abstract 2

A molecular peripheral editing approach based on tetrathiafulvalene molecules is presented to elucidate the structure–property relationship of organic molecules, toward high-performance photodetectors based on organic–graphene hybrids.

## Graphical abstract

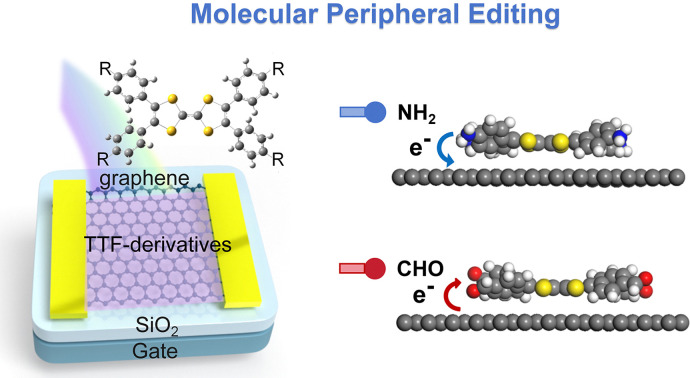

## 1. Introduction

The pioneering work of Geim and Novoselov displaying the outstanding physical properties of graphene has triggered an ever-growing research endeavour targeted at developing fundamental ground-breaking science which has paved the way towards disruptive technological applications in optoelectronics,^1–8^ energy conversion and storage,^9,10^ intelligent flexible electronics,^11,12^ biosensors,^13,14^*etc.* However, despite graphene's intrinsically high charge carrier mobility (2.5 × 10^5^ cm^2^ (V s)^−1^)^15^ and broad-spectrum absorption,^16^ being compelling for the emergence of unprecedented photonics and electronics technologies, its modest light absorption^12,16,17^ and short (∼picosecond) lifetime of the photogenerated hot carriers^18–20^ strongly limited its application in next-generation photodetectors. Thus, protocols to enable controlled “correction” of the optoelectronic properties of graphene are highly sought after.

Molecular functionalization of graphene has emerged as a powerful strategy for modulating graphene's optoelectronic properties.^21–26^ The self-assembly of organic molecules onto the basal plane of graphene can yield a large library of functional hybrid materials, with strong light absorption, tunable energy levels, charge transport properties, and *ad hoc* design properties through fine-tuning of molecular structures.^27,28^ Typical organic sensitizers such as rhodamine,^29,30^ porphyrin,^31^ pentacene,^32,33^ rubrene,^34^ dioctyl-benzothienobenzothiophene (C8-BTBT),^35,36^ covalent organic frameworks (COF_ETBC-TAPT_),^37^ ruthenium complex,^38^ and *N*,*N*′-(4,4′-(1*E*,1′*E*)-2,2-(1,4-phenylene)bis(ethene-2,1-diyl)bis(4,1-phenylene))-bis(2-ethyl-6-methyl-*N*-phenylaniline) (BUBD-1)^39^ have been employed to construct hybrid graphene-containing photodetectors. Yet, the comprehensive understanding of the correlation between the molecular structure and the physical properties of the hybrid toward high-performance organic–graphene hybrid photodetectors remains elusive, especially concerning the role played by the side-groups attached to the functional core of the chosen molecule.

The extended aromatic structure of tetrathiafulvalene (TTF) yields assemblies possessing extended conjugation which endows the architectures with markedly high charge transfer characteristics, high charge carrier mobilities, and large photoresponsivities, making them promising graphene sensitizers for high-performance photodetection.^40,41^ In this work, 4,4′,4′′,4′′′-([2,2′-bi(1,3-dithiolylidene)]-4,4′,5,5′-tetrayl)tetraaniline (TTF–NH_2_) and 4,4′,4′′,4′′′-([2,2′-bi(1,3-dithiolylidene)]-4,4′,5,5′-tetrayl)tetrabenzaldehyde (TTF–CHO) molecules are employed to construct graphene/TTF hybrids by means of simple solution processing. Due to the distinct substituents at the molecular peripheries, significant differences were observed in the modulation of the optoelectronic properties of graphene upon hybrid formation. In view of the electron-donating nature of the substituents, TTF–NH_2_ was found to induce an n-type doping effect when physisorbed onto the basal plane of graphene, whereas TTF–CHO, with its electron-withdrawing substituents, exhibited a p-type doping effect. A comparative analysis revealed that the graphene/TTF–CHO-based photodetectors exhibited a responsivity (*R*) of 1.1 × 10^6^ A W^−1^ and a specific detectivity (D*) of 1.2 × 10^14^ Jones, whereas the graphene/TTF–NH_2_-based photodetectors exhibited an ultra-high responsivity of 1.1 × 10^7^ A W^−1^ and a D* of 6.5 × 10^14^ Jones. Significantly, these figures of merit indicate that our graphene/TTF–NH_2_ hybrid outperforms state-of-the-art graphene-based photodetectors. Moreover, the joint powder X-ray diffraction and theoretical calculations made it possible to ascribe the differences in the responsivities determined for graphene/TTF–NH_2_ and graphene/TTF–CHO hybrids to the differences in the molecular aggregation mode, adsorption energy between molecules and graphene, charge transfer efficiency, and potential differences in interface quality. This work demonstrates that the peripheral editing of organic sensitizers can strongly affect the optoelectronic properties of graphene hybrids, paving the way for the future design of strategies toward high-performance, flexible/wearable graphene-based photodetectors.

## 2. Experimental section

### 2.1 Materials and device fabrication

**Fig. 1:**
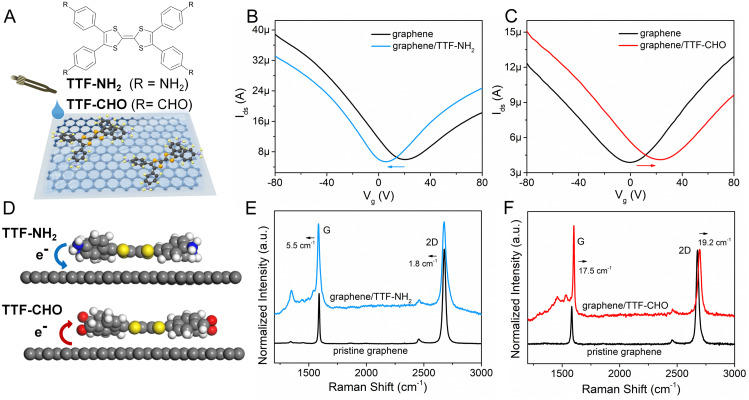
(A) Schematic diagram of physisorption of TTF molecules onto exfoliated graphene. Transfer characteristics of pristine graphene and graphene assembled with (B) TTF–NH_2_ and (C) TTF–CHO in the dark. (D) Schematics of charge transfer between TTF derivatives and the graphene system based on theoretical calculations. Raman spectra of pristine graphene, (E) graphene/TTF–NH_2_ and (F) graphene/TTF–CHO hybrids.

The TTF–NH_2_ and TTF–CHO molecules were purchased from BLDpharm. The scheme in Fig. 1A illustrates the assembly of TTF–NH_2_ or TTF–CHO onto the surface of graphene flakes by the drop-casting method. High-quality graphene flakes were obtained *via* the conventional scotch tape exfoliation method, and were transferred onto SiO_2_ (270 nm)/Si substrates. An atomic force microscopy (AFM) image of high-quality single-layer graphene is shown in Fig. S1 (ESI†). Graphene photodetectors employing back-gated FET device geometry were fabricated *via* a well-established photolithography approach (Microtech laser writer equipped with a 405 nm laser and standard AZ1505 photoresist from Microchemicals). A 50-nm-thick Au film was thermally evaporated onto the patterned substrates, followed by a lift-off process carried out in warm acetone (50 °C). Subsequently, the device was repeatedly rinsed with acetone and isopropanol.

To fabricate the graphene/TTF–NH_2_ hybrid device, 20 μL of TTF–NH_2_ chlorobenzene solution (0.5 mM) was drop-cast onto the graphene field-effect transistors (FETs) on a hot plate at 70 °C in a nitrogen-filled glovebox. The complete evaporation of the solvent was achieved by annealing the device at 160 °C for 2 h. Graphene/TTF–CHO hybrid devices were assembled by either drop-casting or spin-coating by applying a drop of TTF–CHO in chloroform solution (8 mM) onto the graphene-based FET at a spin rate of 600 rpm. Subsequently, the hybrid device was baked at 100 °C for 1 h and then annealed at 160 °C for 2 h.

### 2.2 Electrical measurements

Electrical characterization of the transistors was conducted in a nitrogen-filled glovebox using a probe station equipped with a Keithley 2636A at room temperature. Measurements were carried out in the dark or upon illumination of the device with a monochromator with wavelengths tunable between 300 and 694 nm.

## 3. Results and discussion

The doping of graphene resulting from the electronic interference determined by the physisorbed organic molecules depends on the subtle design of the latter components, which includes their side-groups.^42,43^ We observed significant differences in the doping effect upon physisorption onto the basal plane of graphene TTFs functionalized with either electron-donating amino groups (–NH_2_) or electron-withdrawing aldehyde substituents (–CHO). The schematic diagram in Fig. 1A illustrates the assembly of TTF–NH_2_ or TTF–CHO onto the graphene surface *via* drop casting. To assemble the hybrid structure, 20 μL of a chlorobenzene solution containing TTF-derivatives (0.5 mM) was dropped onto the channel of the graphene-based FET. X-ray photoelectron spectroscopy (XPS) spectra of CVD graphene recorded before and after physisorption of TTF molecules provided evidence for the successful hybrid formation (Fig. S2, ESI†) and complete solvent evaporation upon thermal annealing, as revealed by the rather weak peak intensities of the Cl 2p spectra of graphene/TTF-derivatives. To investigate the doping effect caused by molecular functionalization, the electrical characteristics of the graphene/TTF-derivatives were explored by constructing back-gated FETs. The transfer characteristics of the pristine graphene-based FETs exhibit typical ambipolar behavior. After absorption with TTF–NH_2_, *V*_D_ (corresponding to the Dirac point) shifted from 20 V to 6 V accompanied by an increase in the electron density of 1.12 × 10^12^ cm^−2^, with the electron mobility increasing from 658 cm^2^ (V s)^−1^ to 910 cm^2^ (V s)^−1^, indicating an efficient n-type doping effect. In contrast, upon decoration of the graphene surface with TTF–CHO, *V*_D_ shifted from 0 V to 22 V accompanied by an increase in the hole density of 1.75 × 10^12^ cm^−2^, and the hole mobility increased from 399 cm^2^ (V s)^−1^ to 435 cm^2^ (V s)^−1^, indicating a p-type doping effect. Raman spectroscopy is a non-destructive technique for quantifying the number of layers and exploring the doping effects of graphene by monitoring the intensity ratio (*I*_(2D)_/*I*_(G)_), the positions of the G and 2D bands, and the full width at half-maximum (FWHM) of the G band.^44–47^ The Raman spectra of pristine graphene show an *I*_(2D)_/*I*_(G)_ ratio of approximately 2, indicating a monolayer thickness of the graphene flakes (Fig. 1E and F). Upon physisorption of TTF molecules, the *I*_(2D)_/*I*_(G)_ ratio changed significantly, confirming the occurrence of effective doping. Such doping is also evidenced by a shift of the G peak towards lower and higher wavenumbers for TTF–NH_2_ and TTF–CHO, respectively, suggesting distinct doping effects.^48^ In particular, the interfacing of TTF–NH_2_ on graphene led to a 5.5 cm^−1^ redshift of the G peak (n-type doping), similar to the observations of n-doping effects reported for SWNTs and graphene.^49–52^ Conversely, the position of the G peak shifted by 17.5 cm^−1^ to a higher wavenumber upon interfacing with TTF–CHO (p-type doping). Additionally, this disparity in doping types can also be confirmed by the variation in the secondary-electron cut-off (Fig. S3, ESI†).

**Fig. 2:**
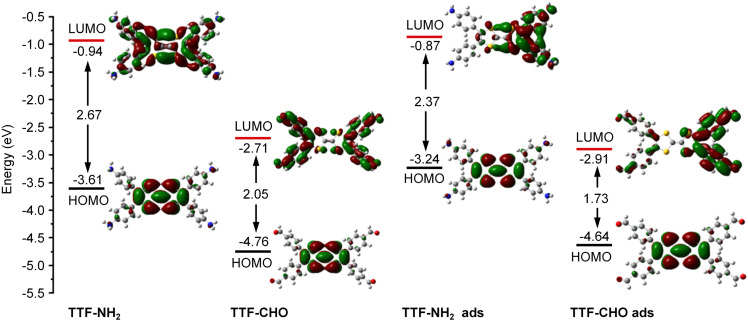
Calculated HOMO and LUMO of TTF–NH_2_ and TTF–CHO monomers before adsorption (TTF–NH_2_, TTF–CHO) and after adsorption (TTF–NH_2_ ads, TTF–CHO ads) onto graphene.

Theoretical calculations were conducted to cast more light on the influence of the interface of TTF-derivatives as a way to tune the electronic properties of graphene. As depicted in Fig. S4 (ESI†), the molecular conformation of the TTF-derivatives before and after adsorption onto graphene was optimized by employing the density functional theory (DFT) calculations. Each central TTF core is connected to four external benzene rings *via* single C–C bonds, with the dihedral angles between the central core and peripheral substituents of 167.54° (for TTF–NH_2_) and 166.03° (for TTF–CHO), respectively. Upon molecular physisorption onto the graphene surface, the molecular planarity is improved through the rotation of single bonds, with dihedral angles of 173.73° and 172.57° for TTF–NH_2_ and TTF–CHO, respectively. This propensity to modify the conformation by adopting a more planar shape facilitates the physical interfacing of the TTF derivatives with the basal plane of graphene through π–π interactions. Additionally, the relative stabilities of the graphene/TTF–NH_2_ and graphene/TTF–CHO hybrids were evaluated through adsorption energy calculations (Table S1, ESI†).^53^ The calculated negative binding energies suggest advantageous adsorption interactions between TTF–NH_2_ and TTF–CHO with graphene, among which the graphene/TTF–NH_2_ system demonstrates more favourable binding. As depicted in Fig. 1D, effective charge transfer occurs between the TTF derivatives and graphene. Consistent with the electrical measurements and Raman spectra analysis, efficient electron transfer takes place from TTF–NH_2_ to graphene, whereas, for the TTF–CHO derivative, electrons transfer from graphene to the molecule. Frontier molecular orbitals were computed using the O3LYP hybrid function with the 6-31G(d) basis set. For the pristine TTF–NH_2_ and TTF–CHO molecules, the highest occupied molecular orbital (HOMO) was confined to the TTF core, whereas the lowest unoccupied molecular orbital (LUMO) was distributed over the whole molecular skeleton (Fig. 2). Interestingly, upon adsorption onto the graphene surface (TTF–NH_2_ ads, TTF–CHO ads), the LUMO undergoes significant changes (located on half of the molecular skeleton), accompanied by a narrower HOMO–LUMO energy gap. This implies effective interfacing between graphene and TTF derivatives *via* π–π interactions, facilitating efficient charge transfer.

By leveraging π–π stacking, the molecular functionalization of graphene facilitates efficient photo-excited charge transfer between molecules and graphene.^54^ Hence, graphene/TTF-derivative hybrid devices hold promise for applications in high-performance photodetectors. To ascertain the detection range of the photodetector, the UV-vis absorption spectra of the light-absorbing materials (TTF–NH_2_ and TTF–CHO) were recorded (Fig. S5, ESI†). The TTF–NH_2_ and TTF–CHO solutions exhibit absorption across the UV and visible light regions (250–600 nm), with the maximum absorption peaks centred at approximately 300 nm. The absorbance at 300 nm closely complies with the Beer–Lambert behavior when plotted as a function of the concentration. Moreover, TTF–CHO solutions exhibit superior light absorption compared to TTF–NH_2_ at equivalent concentrations. To evaluate the absorption characteristics of the graphene/TTF-derivative hybrids, the UV-vis absorption spectra of pristine CVD-graphene, TTF-derivative films, and their hybrids are illustrated in Fig. S6 (ESI†). Notably, pristine CVD-graphene exhibits minimal light absorption, with primary absorption contributions in the hybrids originating from TTF derivatives. Hence, the optoelectronic properties of graphene/TTF-derivatives were investigated by illuminating the hybrids at wavelengths of 300 nm, 450 nm, 550 nm, and 600 nm.

**Fig. 3:**
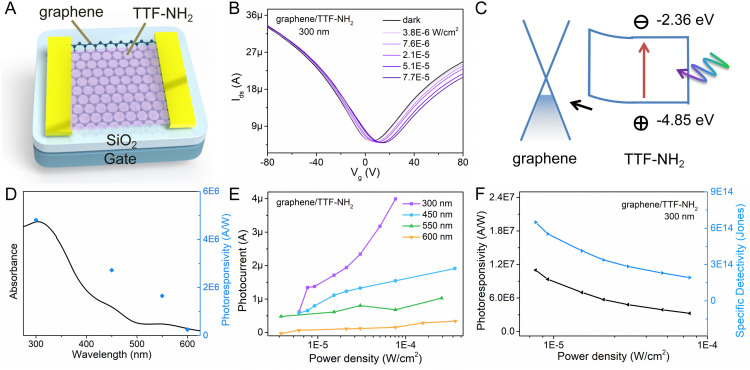
(A) Schematics of the device structure of graphene/self-assembled TTF–NH_2_. (B) *I*_ds_ − *V*_g_ characteristics of the hybrid phototransistor based on graphene/TTF–NH_2_ in the dark and different optical illumination power densities at a 300 nm signal (*V*_ds_ = 50 mV). (C) Energy diagram of the graphene/TTF–NH_2_ device. (D) UV-vis absorption spectra of graphene/TTF–NH_2_ and the corresponding photoresponsivity at a selected wavelength (*V*_g_ = 36 V, power density = 3.0 × 10^−5^ W cm^−2^). (E) Variations in the photoresponsivity with different optical illumination power densities and wavelengths. (F) Photoresponsivity and specific detectivity change with different incident power densities of 300 nm light.

The geometry of the photodetector device is displayed in Fig. 3A. To gain insight into the impact of molecular functionalization on the performance of graphene-based photodetectors, we investigated the photoresponse of the pristine graphene devices. Fig. S8 (ESI†) reveals the absence of a photoresponse from the graphene photodetector under illumination at various wavelengths, primarily due to the low absorption coefficient of graphene. Fig. 3 portrays the photo-response characteristics of the graphene device functionalized with TTF–NH_2_ molecules. Fig. 3B shows the drain current as a function of the gate voltage (*V*_g_) for the graphene/TTF–NH_2_ hybrid photodetector in the dark and varying 300 nm illumination power densities, with a constant source-drain voltage (*V*_ds_) of 50 mV. With higher illumination power densities under 300 nm light irradiation, the Dirac point incrementally shifts towards positive voltage values, accompanied by an increase in the photocurrent. Even under an extremely low illumination power density (7.6 × 10^−6^ W cm^−2^), significant photocurrent values can still be observed. Moreover, the photocurrent displays a strong modulation behavior with respect to the gate voltage, exhibiting a negative photocurrent at *V*_g_ > *V*_D_ and a positive photocurrent at *V*_g_ < *V*_D_. In order to ascertain the band positions of the Frontier orbitals of organic molecules and further explore the mechanism of photodetection in hybrid devices, cyclic voltammetry (CV) studies were conducted on the TTF derivatives (Fig. S9, ESI†). They revealed a HOMO energy level of TTF–NH_2_ corresponding to −4.85 eV. The LUMO energy level was quantified by integrating the HOMO energy level with the optical energy bandgap, and was calculated as −2.36 eV. Upon molecular functionalization of graphene with TTF–NH_2_, n-type doping effects are observed, indicating the migration of holes from graphene to the organic layer, thereby establishing an interfacial built-in electric field at the graphene/TTF–NH_2_ interface (Fig. 3C). Under illumination, electrons within the light-absorbing layer (TTF–NH_2_) undergo transitions from the HOMO to the LUMO, thereby generating electron–hole pairs. Such photogenerated electron–hole pairs can be separated by the built-in interfacial electric field, with holes injected from TTF–NH_2_ into graphene. The high photoconductive gain of hybrid photodetectors can be attributed to the photogating effect, wherein the accumulated electrons in TTF–NH_2_ establish a local electric field to gate the graphene, enabling the shift of *V*_D_ to higher gate voltages and efficient photon detection.^54,55^ Benefiting from the picosecond-scale transit time of the carriers in graphene, the recombination between electrons and holes in the light-absorbing layer is hindered.^56^ When holes dominate as the primary charge carriers in graphene (*V*_g_ < *V*_D_), photo-generated holes are injected from TTF–NH_2_ into graphene, leading to the accumulation of holes within the graphene channel and subsequently generating a positive photocurrent. Conversely, when electrons serve as the primary charge carriers in graphene (*V*_g_ > *V*_D_), the injection of photogenerated holes from the TTF–NH_2_ layer and the photogating effect leads to a reduction in the electron concentration within the graphene, resulting in a negative photocurrent. Due to the modulation of the Fermi level of graphene by the gate voltage, a higher photocurrent is exhibited in the positive gate region. The relationship between the photocurrent and illumination power densities at different wavelengths exhibits a wide linear dynamic range, particularly at 300 nm and 450 nm, demonstrating the potential of graphene/TTF–NH_2_ for photodetection in the ultraviolet and visible light regions (Fig. 3E). Subsequently, key figures of merit of the photodetector, including responsivity and specific detectivity, were evaluated.^57^ As depicted in Fig. 3D, the photoresponsivity, as a function of the incident wavelength, consistently fits well with the absorption curve of the graphene/TTF–NH_2_ hybrid. As the graphene/TTF–NH_2_ hybrid photodetector exhibits maximum photoresponsivity at 300 nm, the factors *R* and D* were plotted as a function of the incident illumination power densities under 300 nm irradiation (Fig. 3F). At the lowest power density, this hybrid device exhibits an ultra-high photoresponsivity of 1.1 × 10^7^ A W^−1^ and a specific detectivity of 6.5 × 10^14^ Jones, outperforming state-of-the-art graphene-based photodetectors.^37,56,58,59^ More devices based on graphene/TTF–NH_2_ were fabricated to evaluate the photo-response of the hybrid photodetectors (Table S3 and Fig. S10, ESI†). Within the range of irradiation power densities analysed, the maximum photoresponsivities were recorded as 1.2 × 10^6^ A W^−1^, 1.8 × 10^7^ A W^−1^, and 2.1 × 10^7^ A W^−1^, respectively. Moreover, the graphene/TTF–NH_2_ device still exhibits ultra-high photoresponsivity of 1 × 10^7^ A W^−1^ after 100 days of storage (Fig. S13, ESI†). This implies that achieving a high photoresponsivity based on the graphene/TTF–NH_2_ hybrid through molecular functionalization is feasible.

**Fig. 4:**
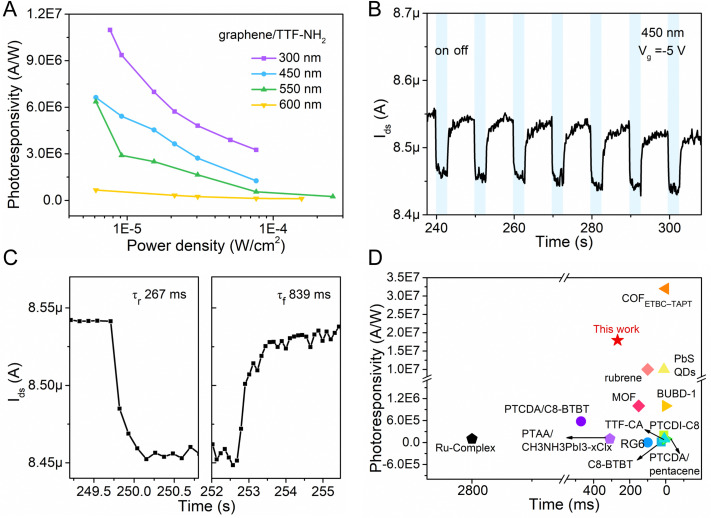
(A) Photoresponsivity of the graphene/TTF–NH_2_ transistor at different wavelengths. (B) and (C) Temporal photocurrent of the graphene/TTF–NH_2_ transistor under dark and 450 nm irradiation conditions, and the corresponding transfer curves are shown in Fig. S10B (ESI†). (D) Comparison of the photoresponsivity and response time performance of this work (graphene/TTF–NH_2_) with reported graphene-based photodetectors.

The dynamic photoresponse of the hybrid device was further evaluated, and it demonstrated good stability over multiple cycles (Fig. 4B). As shown in Fig. 4C, the time-dependent photocurrent response indicates a rise time of ∼267 ms (calculated at 10–90% of the maximum value) and a fall time of 839 ms. The lower recovery time could be attributed to the disorders in the organic film, which trap photogenerated electrons and prolong the annihilation process of photo-generated electron–hole pairs.^36^ Compared with other reported graphene-based hybrid photodetectors, the graphene/TTF–NH_2_ device exhibits superior overall performance, and we anticipate that the response times can be improved by further optimizing the interface (Fig. 4D and Table S2, ESI†).^29,34,37–39,56,60–66^

The photoresponse characteristics of the graphene/TTF–CHO hybrid devices are also explored and plotted in Fig. S11 and S12 (ESI†). The photoresponse mechanism of graphene/TTF–CHO is the same as that of graphene/TTF–NH_2_. Under illumination, photogenerated holes are injected from the organic layer into the graphene channel, and the Dirac point is observed to shift toward a positive gate voltage. Similarly, the photoresponsivity of the graphene/TTF–CHO hybrids also shows a pronounced dependence on the optical wavelength. Despite employing different solution processing methods (spin-coating method shown in Fig. S11 and drop-casting method shown in Fig. S12, ESI†), both devices exhibit similar photoresponsivities, evaluated as 2.0 × 10^6^ A W^−1^ and 1.1 × 10^6^ A W^−1^, respectively. The graphene/TTF–CHO hybrid photodetectors also display ultra-high specific detectivities, reaching 3.2 × 10^14^ Jones and 1.2 × 10^14^ Jones, respectively. Despite exhibiting higher absorbance compared to TTF–NH_2_ at the same concentration, the graphene/TTF–CHO hybrid devices show lower photo-responsivity. Besides, the structural characteristics of the organic films were investigated using powder X-ray diffraction (PXRD) to explore the possible factors affecting the photoresponse. The TTF–NH_2_ and TTF–CHO solutions are drop-cast onto freshly cleaved HOPG surfaces under identical experimental conditions. As shown in Fig. S7 (ESI†), the strong diffraction peak observed at 24.02° could be attributed to the HOPG signal. The strong diffraction peak at an angle of 6.07° indicates that the TTF–CHO film is highly crystalline, whereas TTF–NH_2_ exhibits an amorphous structure. The differences depicted in the PXRD patterns imply that the TTF–NH_2_ and TTF–CHO molecules possess distinct aggregation modes within the organic film. These results might suggest the molecular interactions between graphene and TTF–NH_2_ are stronger than the tendency of TTF–NH_2_ to undergo aggregation, which will lead to a more effective charge transfer and, thus, a better device performance compared with TTF–CHO.^30,54^

Additionally, the thickness-dependent photoresponse behavior was examined by studying the increased absorption layers of TTF-derivatives (Fig. S14 and S15, ESI†). As the film thickness increases, the Dirac point exhibits a more pronounced shift. TTF–NH_2_ shifts towards a negative voltage (from 20 V to −25 V), while TTF–CHO shifts towards a positive voltage (from 6 V to 44 V). The photoresponse was found to depend on the amount of semiconductor.^67^ In the case of TTF-derivatives, the photoresponse decays as the thickness of the absorption layer increases. This indicates that the thinner films of TTF-derivatives have a higher charge separation efficiency, owing to the limited exciton diffusion lengths of organic semiconductors.^27^ Based on the above studies, we attribute the high photoresponse of TTF-derivatives to these main factors: highly absorptive organic layers, strong interface coupling, suitable band alignment, and efficient charge separation.

## 4. Conclusions

In summary, fine control over the molecular functionalization made it possible to generate new hybrids based on graphene/TTF-derivatives by means of simple solution processing methods. The peripheries of the TTF cores were decorated with electron-donating (NH_2_) or electron-withdrawing (CHO) substituents. The controlled physisorption of TTF–NH_2_ or TTF–CHO molecules onto the basal plane of graphene yielded n-type or p-type doping effects. Benefiting from π–π interactions, effective charge transfer occurs between the photoactive functional components and graphene. The graphene/TTF–NH_2_ hybrid photodetector exhibits an ultra-high responsivity of 1.1 × 10^7^ A W^−1^ and a specific detectivity of 6.5 × 10^14^ Jones, demonstrating an exceptionally high-performance compared to reported photodetectors. Furthermore, differences in substituents at the periphery would influence the band alignment, molecular aggregation mode, adsorption energy between molecules and graphene, and the efficiency of charge transfer within the hybrid system. Considering the aforementioned factors and potential differences in the interface quality, the graphene/TTF–CHO hybrid exhibits a slightly lower photoresponsivity (1.1 × 10^6^ A W^−1^). These results not only demonstrate the effectiveness of the employed peripheral editing approach in optimizing the optoelectronic characteristics of graphene but also hold tremendous potential for future high-performance, flexible/wearable photodetection applications.

## Author contributions

S. D., P. S., and Z. L. conceived and designed the project. S. D. and C. W. performed the FET device fabrication and characterization. M. X., Y. W., and L. C. conducted DFT calculations. S. D. and B. H. performed Raman spectroscopy and XPS. S. D. and S. X. performed PXRD measurements. K. W. performed the CV measurements. S. D. and A. Z. conducted the STM study. B. X. and W. T. co-supervised molecular selection and photophysics processes of the device. S. D. wrote the manuscript with comments and suggestions from all co-authors.

## Data availability

The data supporting this article have been included as part of the ESI.†

## Conflicts of interest

There are no conflicts to declare.

## Supplementary Material

TC-012-D4TC02010C-s001
