# Supplementary material for: Highly efficient organic–graphene hybrid photodetectors via molecular peripheral editing
Source: J Mater Chem C Mater. 2024 Aug 12;12(36):14667–74. doi: 10.1039/d4tc02010c (PMC11515839; doi:10.1039/d4tc02010c)
Supplement: TC-012-D4TC02010C-s001 [file TC-012-D4TC02010C-s001.pdf]

Supplementary Information (SI) for Journal of Materials Chemistry C.
This journal is © The Royal Society of Chemistry 2024

Supporting Information

Highly Efficient Organic-Graphene Hybrid Photodetectors *via* Molecular Peripheral Editing

Shuting Dai, [a,b] Miao Xie, [c] Can Wang, [a] Yuying Wang, [c] Bin Han, [b] Shunqi Xu, [b] Kexin Wang, [b,d] Anna Zhuravlova, [b] Bin Xu, [a] Lifeng Chi, [c] Wenjing Tian, [a] Paolo Samorì,*[b] Zhaoyang Liu*[a]

[a] State Key Laboratory of Supramolecular Structure and Materials, Jilin University

Changchun 130012, China

[b] ISIS, Université de Strasbourg and CNRS, 8 allée Gaspard Monge

Strasbourg 67000, France

[c] Institute of Functional Nano & Soft Materials (FUNSOM), Soochow University

199 Renai Road, Suzhou 215123, China

[d] Key Laboratory of Biomass Chemical Engineering of Ministry of Education, College of Chemical and Biological Engineering, Zhejiang University

Hangzhou 310027, China

* Corresponding authors. Emails for correspondence: samori@unistra.fr (Paolo Samorì), zhaoyangliu@jlu.edu.cn (Zhaoyang Liu)

Methods

1. Measurements

UV-vis absorption spectra

The UV/vis absorption spectra of the TTF-NH_2 and TTF-CHO solutions, as well as the TTF-Graphene hybrid films supported on the quartz substrate were recorded with a JASCO V-650 spectrophotometer.

Raman Spectroscopy

Raman spectroscopy investigations were carried out in ambient conditions with a Renishaw inVia spectrometer equipped with a 532 nm laser, and the wavenumber (energy) resolution is about 1.25 cm^{-1} (≈1 meV). The excitation power was kept lower than 1 mW to avoid local heating damage effects.

XPS Measurements

XPS measurements were carried out with a Thermo Scientific K-Alpha X-ray photoelectron spectrometer with Al anode as the X-ray source (X-ray radiation of 1486 eV, Spot sizes of 400 μm). And basic chamber pressure is about 10^{-9} mbar. All XPS spectra were calibrated using the C 1s peak at 284.8 eV as a reference.

2. Equations for the calculation of parameters in devices

2.1 Mobility

$$\mu = \frac{L}{W C_i V_{ds}} \frac{\partial I_{ds}}{\partial V_G}$$

2.2 Charge in the carrier density change

$$\Delta n = \frac{C_i \Delta V_D}{e} = 7.99 \times 10^{10} \Delta V_D \text{ cm}^{-2}$$

2.3 Photoresponsivity

$$R = \frac{I_{ph}}{P_{in}}$$

2.4 Specific Detectivity

$$D^* = \frac{\sqrt{AB}}{NEP} = \frac{R}{\sqrt{\frac{2eI_{dark}}{A}}}$$

3. Computational Details

The quantum mechanics calculations were performed using the Vienna Ab initio Simulation Package (VASP) version 5.4.4,[1, 2] employing the projector augmented wave (PAW) method[3, 4] and a plane wave basis set. We utilized the Perdew, Burke, and Ernzerhof (PBE)[5] flavor of Density Functional Theory (DFT), supplemented by the post-stage DFT-D3 method to account for London dispersion (van der Waals attraction) with Becke-Johnson damping. The PAW method effectively addressed core-valence interactions. A kinetic energy cutoff of 400 eV was applied for plane wave expansions, and reciprocal space was sampled using the Γ-centered Monkhorst-Pack scheme with a grid of 1 × 1 × 1. Convergence criteria were judiciously set at 1×10^{-5} eV for energy differences in solving the electronic wave function, while atomic coordinates were diligently converged to within 1×10^{-2} eV $Å^{-1}$ for maximal force components.

The gas-phase electronic structure calculations were performed using the Gaussian 16 suite of programs (Revision A.01).[6] The geometrical structures of the ground-state molecules were optimized by using the B3LYP hybrid functional combined with the 6-31G(d) basis. Compared with the experimental data, it was shown that the energy levels obtained by O3LYP hybrid function is more accurate. Hence, the molecular orbital diagrams were calculated at the O3LYP/ 6-31G(d) level.

In our investigation, we rigorously modeled the graphene surface using 14×14 unit cells, incorporating a 20 Å vacuum thickness to obviate any potential interactions between adjacent slabs. Throughout our calculations, all atomic entities were allowed to relax, thereby ensuring the attainment of a stable configuration.

Table S1. Binding energies (eV) between TTF-derivatives and graphene system.

**Graphene/TTF-NH_2**	**TTF-NH_2**	**graphene**	**Binding energy[a]**
-3093.97	-409.24	-2682.48	-2.25
**Graphene/TTF-CHO**	**TTF-CHO**	**graphene**	**Binding energy**
-3106.73	-422.12	-2682.48	-2.13

[a] binding energy $=E_{total}-E_{graphene}-E_{TTF}$, where E_{total}, $E_{graphene}$, and E_{TTF} represent the calculated energies of the total graphene/TTF-derivatives system, graphene and TTF derivatives, respectively.

Table S2. The figures of merit of several reported graphene-based hybrid, graphene/TTF-NH_2, and graphene/TTF-CHO photodetectors.

	**Responsivity (A/W)**	**Detectivity (Jones)**	**Response time**	**References**
RG6	460	10^{10}	<100 ms	[7]
C8-BTBT	1.57×10^4	-	<25 ms	[8]
Ru-Complex	1×10^5	-	2.8 s	[9]
$COF_{ETBC-TAPT}$	3.2×10^7	6×10^{13}	1.14 ms	[10]
PTCDA/pentacene	10^5	-	<30 μs	[11]
PTCDI-C8	2×10^5	10^{16}	~10 ms	[12]
rubrene	10^7	9×10^{11}	~100 ms	[13]
MOF	$>10^6$	6.9×10^{14}	<150 ms	[14]
TTF-CA	10^5	10^{13}	8 ms	[15]
PTCDA/C8-BTBT	5.76×10^5	-	470 ms	[16]
PTAA/ CH3NH3PbI3−xCl x	10^5	10^{13}	310 ms	[17]
PbS QDs	10^7	7×10^{13}	10 ms	[18]
BUBD-1	$\sim10^6$	$>10^{12}$	0.3 ms	[19]
TTF-NH_2	1.8×10^7	1.1×10^{15}	267 ms	This work
TTF-CHO	2.0×10^6	3.2×10^{14}	381 ms	This work

Table S3. The figures of merit of graphene/TTF-NH_2 and graphene/TTF-CHO photodetectors under 300 nm irradiation.

**Graphene/ TTF-NH_2**	Power density (W/cm^2)	7.6E-6	9.2E-6	1.5E-5	2.1E-5	3.0E-5
	Responsivity (A/W)	1.1E7	9.4E6	7.0E6	5.7E6	4.8E6
	Detectivity (Jones)	6.5E14	5.5E14	4.1E14	3.4E14	2.8E14
**Graphene/ TTF-NH_2**	Power density (W/cm^2)	6.7E-6	9.0E-6	1.7E-5	4.6E-5	1.1E-4
	Responsivity (A/W)	1.2E6	1.09E6	8.7E5	4.3E5	1.3E5
	Detectivity (Jones)	1.5E14	1.3E14	1.1E14	5.3E13	1.6E13
**Graphene/ TTF-NH_2**	Power density (W/cm^2)	1.1E-6	6.1E-6	7.6E-6	9.2E-6	1.5E-5
	Responsivity (A/W)	1.8E7	1.3E7	1.2E7	1.1E7	8.0E6
	Detectivity (Jones)	1.1E15	7.6E14	7.0E14	6.5E14	4.8E14
**Graphene/ TTF-NH_2**	Power density (W/cm^2)	3.8E-6	7.6E-6	9.2E-6	1.5E-5	2.1E-5
	Responsivity (A/W)	2.1E7	1.7E7	1.6E7	1.3E7	1.1E7
	Detectivity (Jones)	1.1E15	9.6E14	8.8E14	7.2E14	5.8E14
**Graphene/ TTF-CHO**	Power density (W/cm^2)	3.8E-6	7.6E-6	1.5E-5	2.1E-5	3.0E-5
	Responsivity (A/W)	2.0E6	1.5E6	1.0E6	9.6E5	7.8E5

	Detectivity (Jones)	3.2E14	2.4E14	1.7E14	1.5E14	1.3E14
Graphene/ TTF-CHO	Power density (W/cm^2)	3.8E-6	7.6E-5	1.5E-5	2.1E-5	3.0E-5
	Responsivity (A/W)	1.1E6	9.4E5	8.3E5	7.0E5	6.2E5
	Detectivity (Jones)	1.2E14	1.0E14	9.2E13	7.7E13	6.8E13

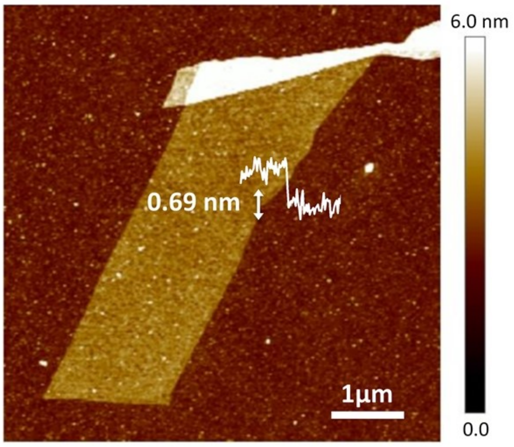

Figure S1. Monolayer graphene visualized by AFM, showing a thickness of 0.69 nm.

The AFM measurement of the selected graphene flake for device fabrication shows a thickness of 0.69 nm, demonstrating the monolayer nature of graphene.

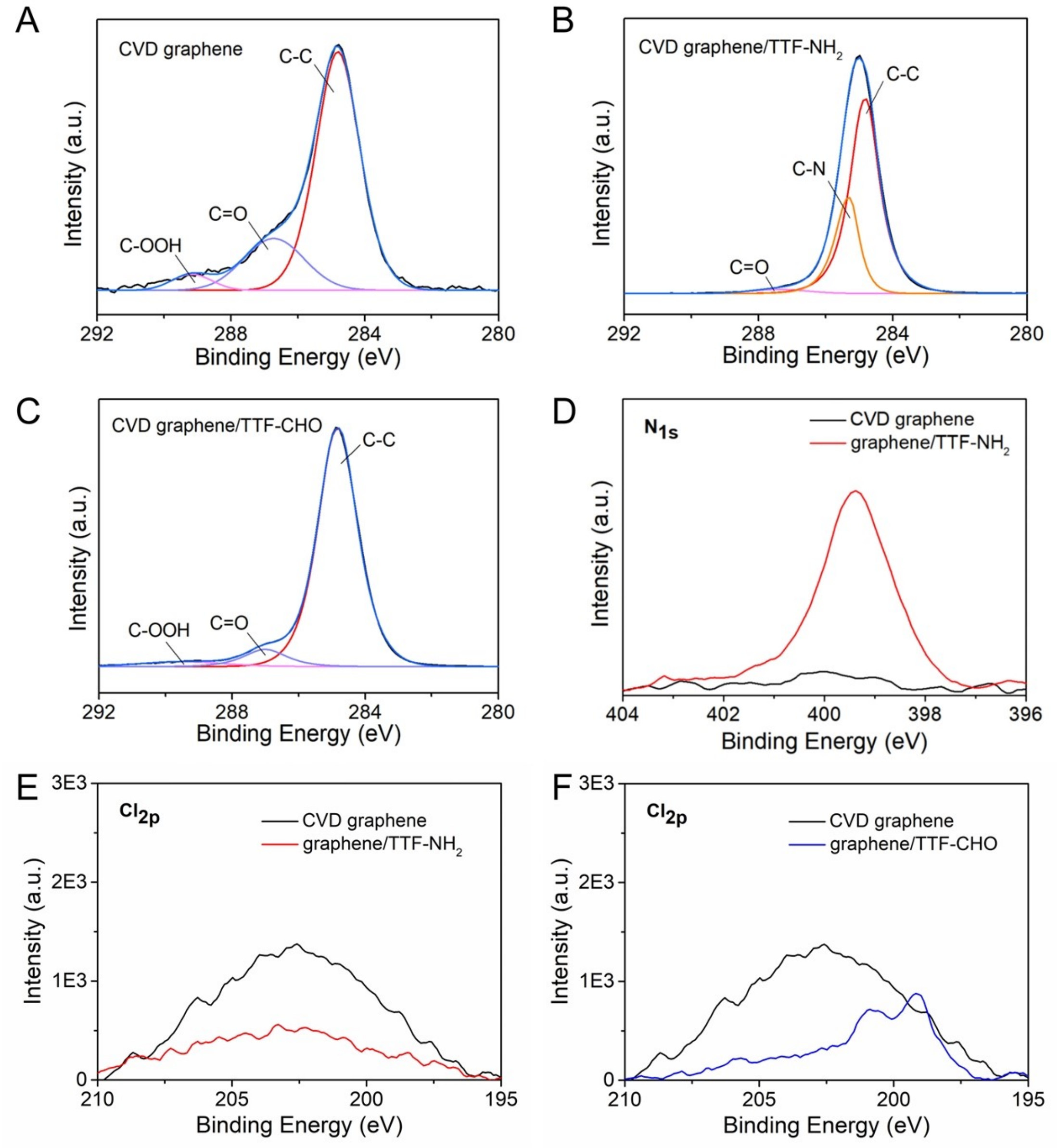

Figure S2. C1s XPS spectra of (A) CVD graphene, (B) CVD graphene/TTF-NH_2 hybrids, and (C) CVD graphene/TTF-CHO hybrids; (D) N1s XPS spectra of CVD graphene/TTF-NH_2 hybrids; Cl2p XPS spectra of (E) CVD graphene/TTF-NH_2 hybrids and (F) CVD graphene/TTF-CHO hybrids.

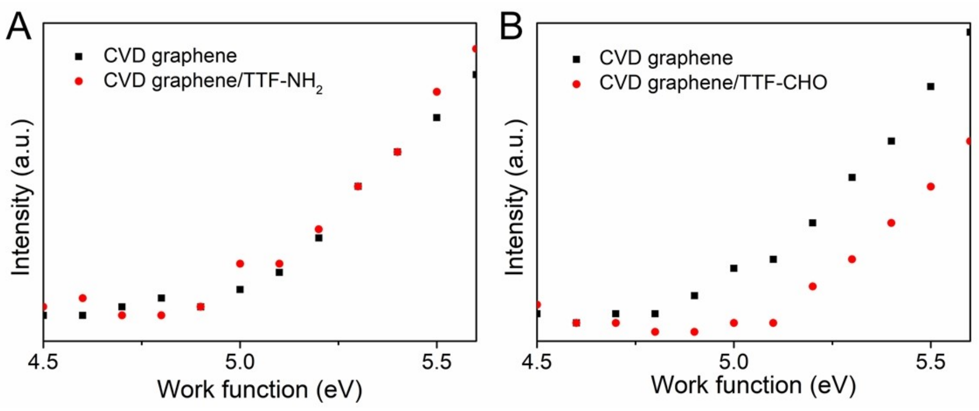

Figure S3. Work function plots of (A) CVD graphene/TTF-NH_2 and (B) CVD graphene/TTF-CHO measured by Photoelectron Yield Spectroscopy in Ambient conditions (PYSA).

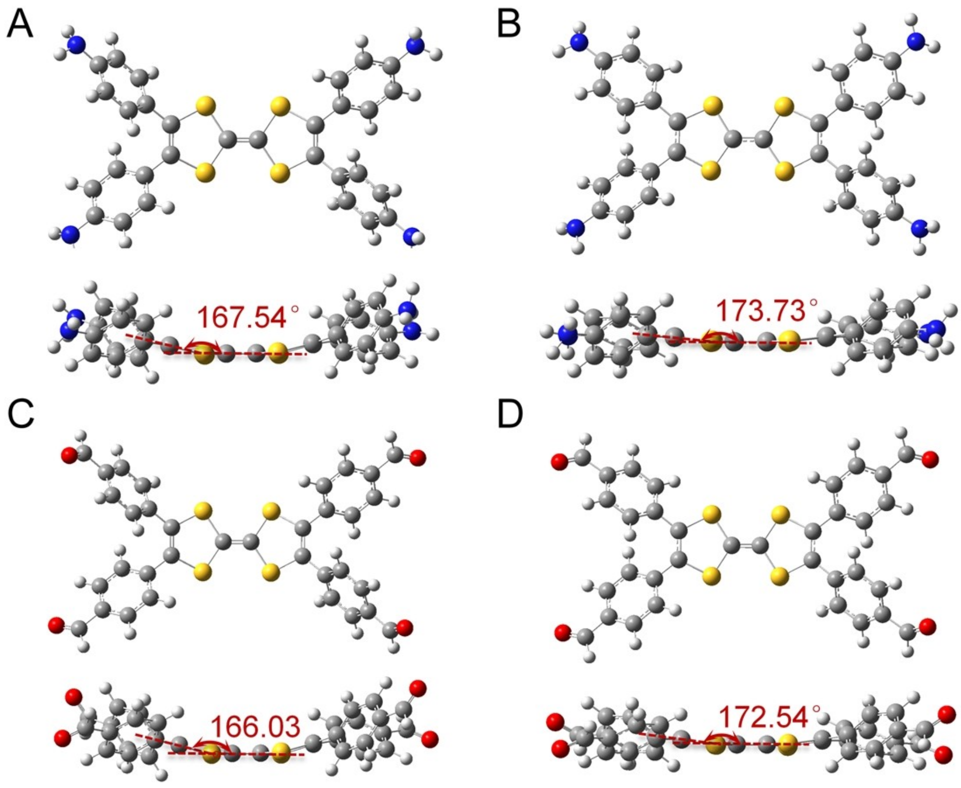

Figure S4. Molecular configurations calculated by density functional theory (DFT) of pristine (A) TTF-NH_2 and (C) TTF-CHO; Optimized molecular configurations (i.e., configurations with the lowest energy) of (B) TTF-NH_2 and (D) TTF-CHO when adsorbed onto the graphene surface.

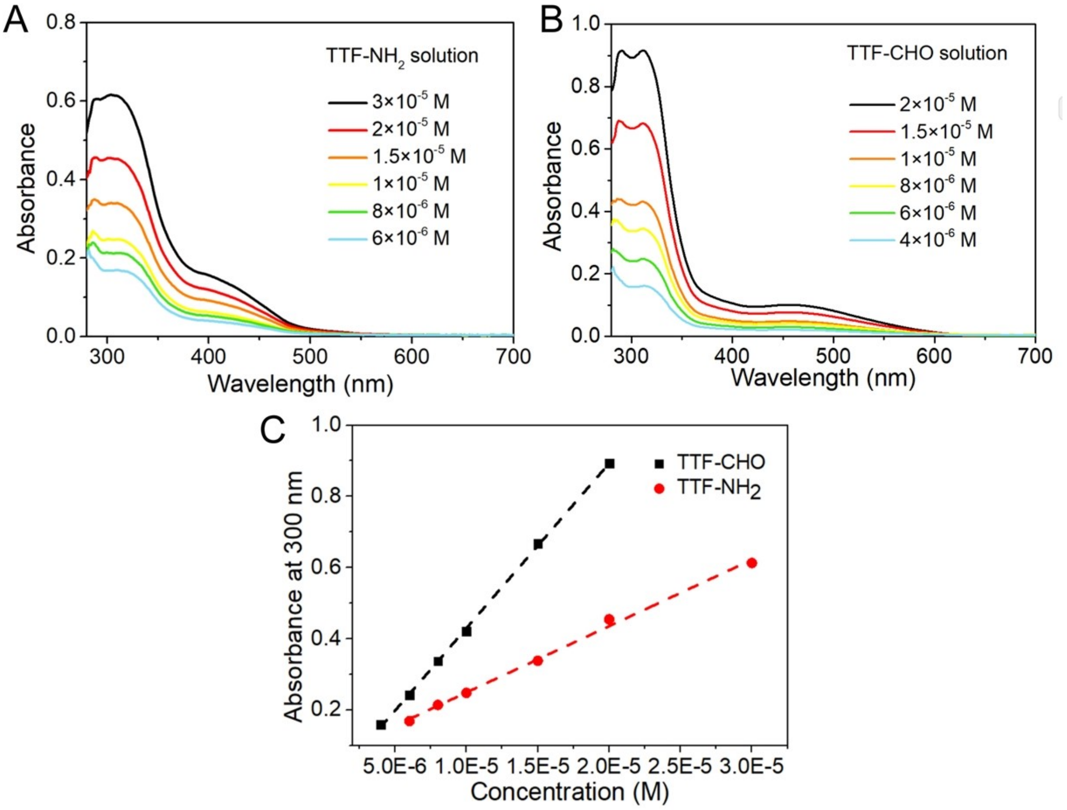

Figure S5. Normalized UV-Vis absorption spectra of (A) TTF-NH_2 and (B) TTF-CHO solution with different concentrations; (C) Absorbance at 300 nm as a function of the concentration of TTF-NH_2 (R^2 = 0.9956) and TTF-CHO (R^2 = 0.9995) solutions.

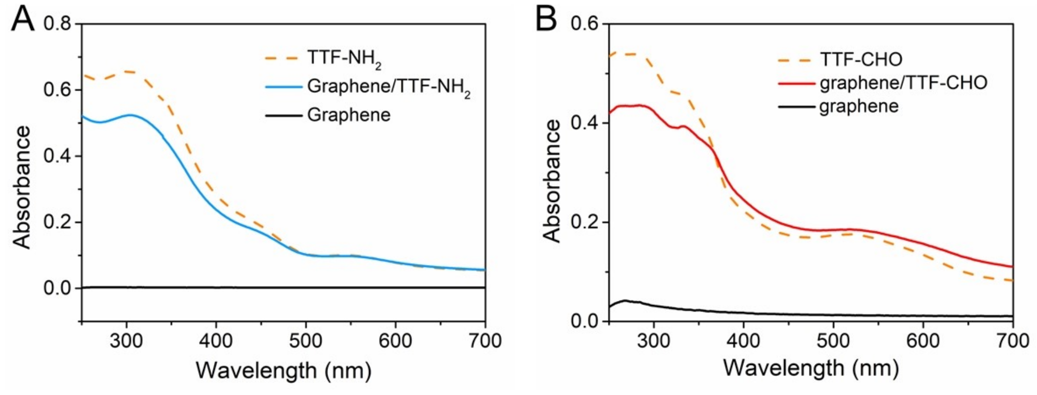

Figure S6. (A) UV-vis absorption spectra of pristine CVD-graphene, TTF-NH_2, and graphene/TTF-NH_2. (B) UV-vis absorption spectra of pristine CVD-graphene, TTF-CHO, and graphene/TTF-CHO.

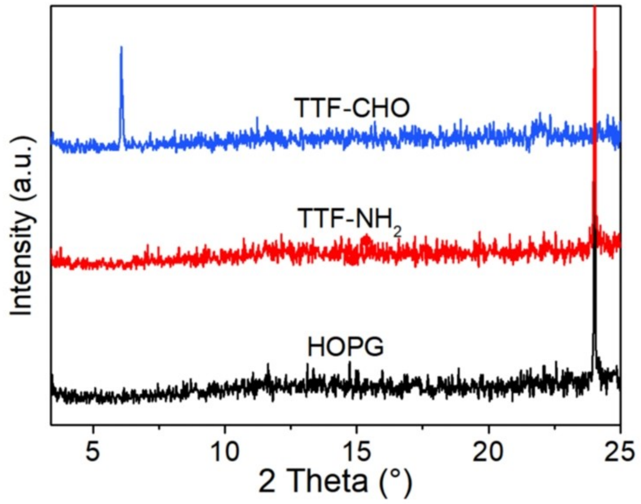

Figure S7. PXRD patterns of pristine HOPG and TTF-derivatives drop-casted onto the HOPG surface.

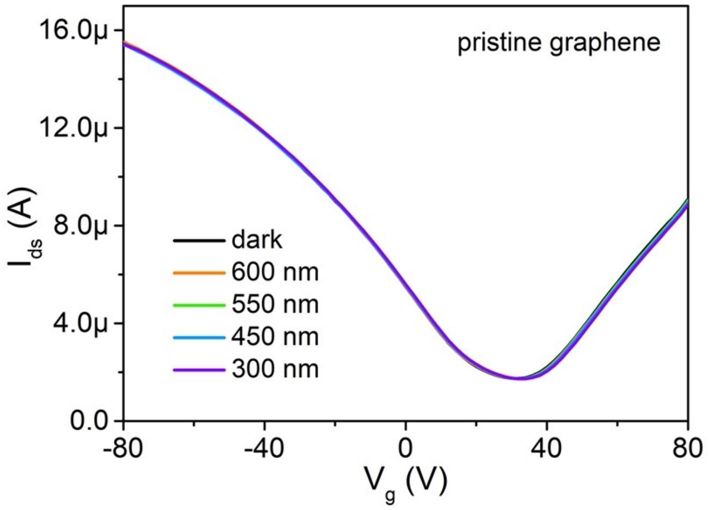

Figure S8. Transfer characteristics of the pristine graphene FET under dark and light irradiation with different wavelength.

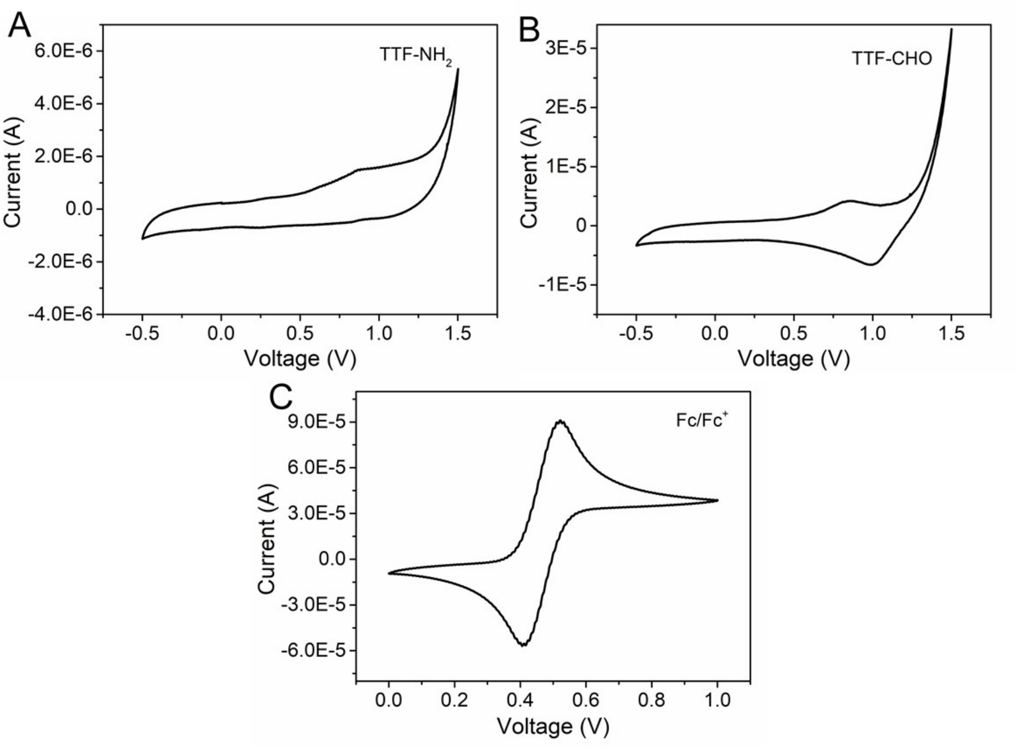

Figure S9. Cyclic voltammetry curves of (A) TTF-NH_2, (B) TTF-CHO, and (C) ferrocenium/ferrocene.

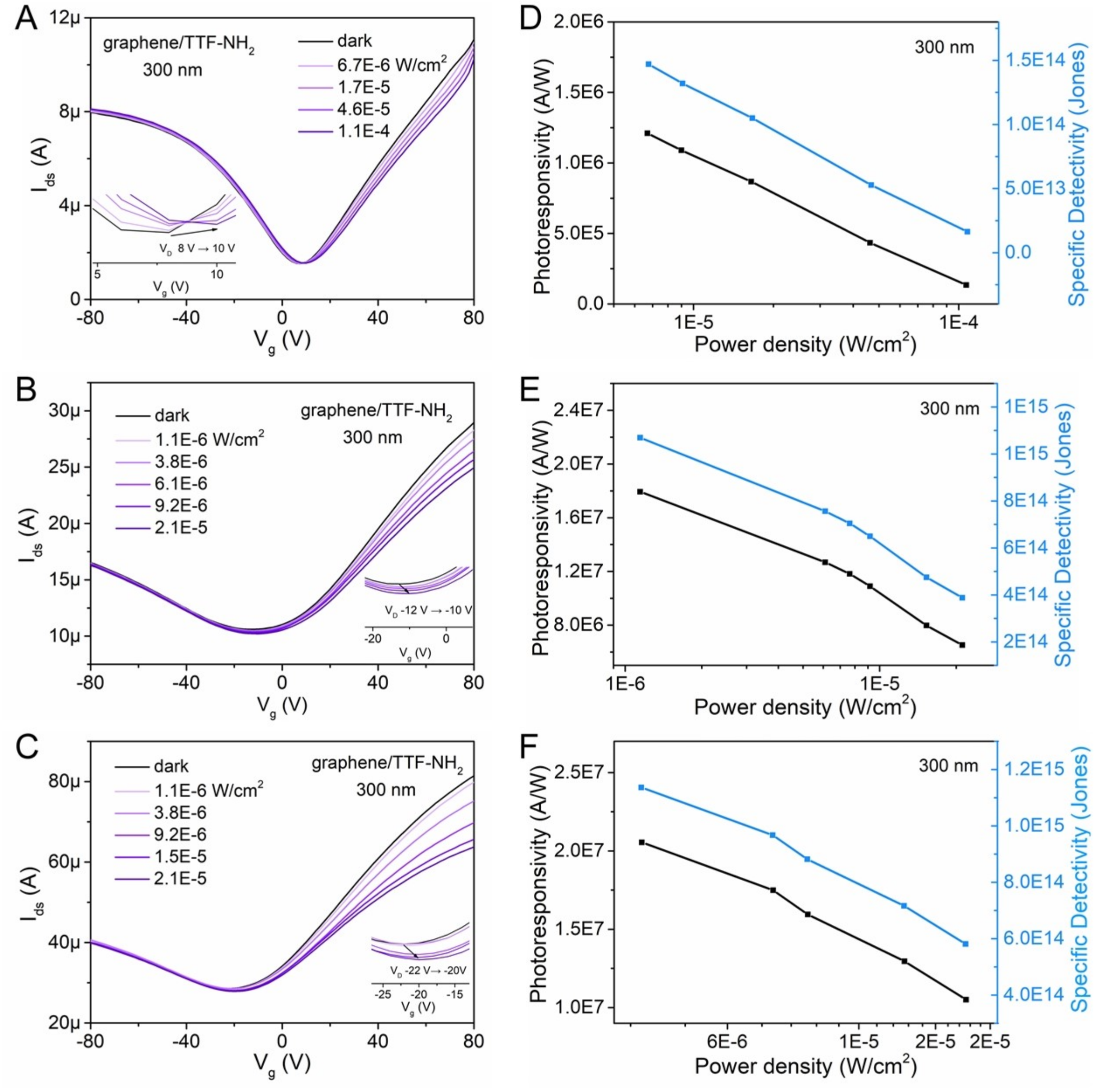

Figure S10. (A-C) Transfer curves of different graphene/TTF-NH_2 hybrid devices under light illumination (V_{ds}=50 mV); (D-F) Corresponding photoresponsivity and specific detectivity change in response to 300 nm illumination under varying power densities.

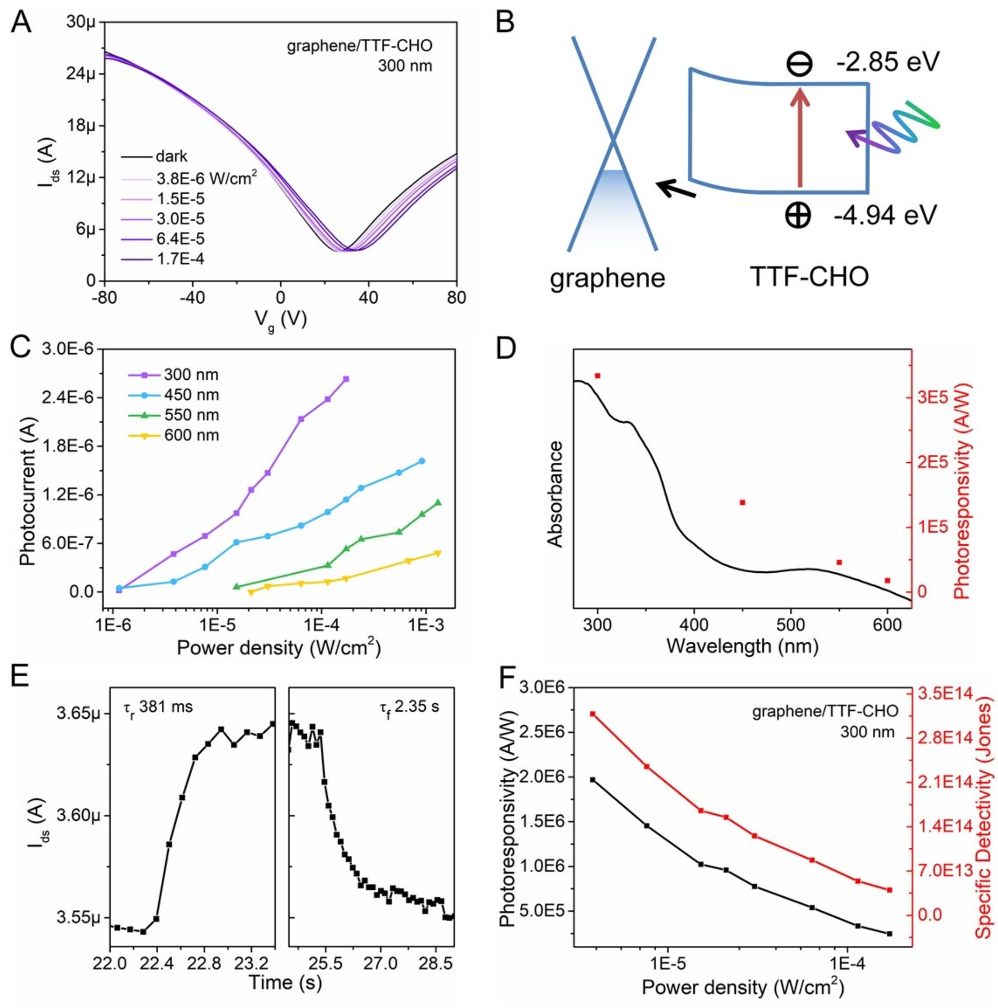

Figure S11. (A) Transfer curves of graphene/TTF-CHO hybrid device, where TTF-CHO was spin-coated onto the graphene FET, under different power densities of 300 nm illumination (V_{ds}=50 mV); (B) Energy diagrams of the graphene/TTF-CHO hybrids; (C) Photocurrent curves with varying illumination power densities and wavelengths; (D) Photoresponsivity of graphene/TTF-CHO transistor under different wavelengths. The black line is the corresponding absorbance of the TTF-CHO film; (E) Temporal photocurrent response of the device under dark and 450 nm irradiation; (F) Photoresponsivity and specific detectivity change in response to 300 nm illumination under varying power densities.

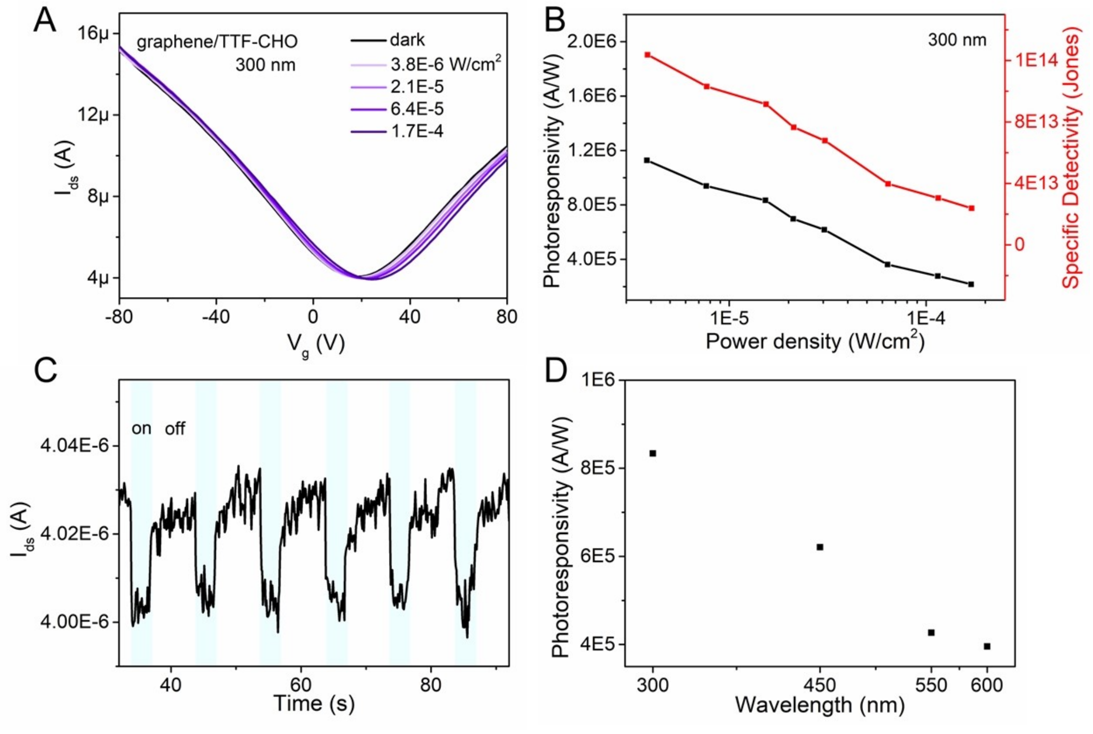

Figure S12. (A) Transfer curves of graphene/TTF-CHO hybrid device, where TTF-CHO was drop-cast onto the graphene FET at a concentration of 0.5 mM at 70 °C, under different power densities of 300 nm illumination (V_{ds} = 50 mV); Corresponding (B) photoresponsivity and specific detectivity performance under 300 nm illumination, (C) temporal photocurrent under dark and 450 nm irradiation, and (D) photoresponsivity under different wavelengths of the graphene/TTF-CHO transistor.

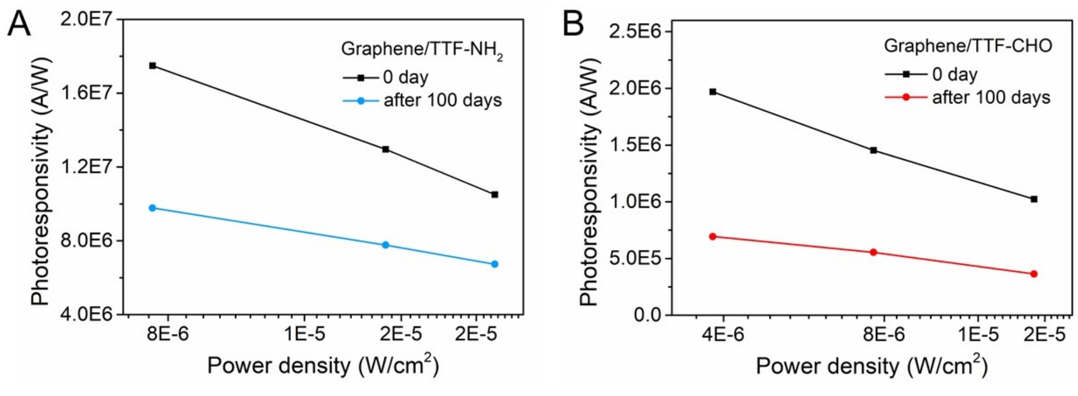

Figure S13. Long-term stability tests based on the photoresponsitivity of graphene/TTF-NH_2 (A) and graphene/TTF-CHO (B) hybrid devices.

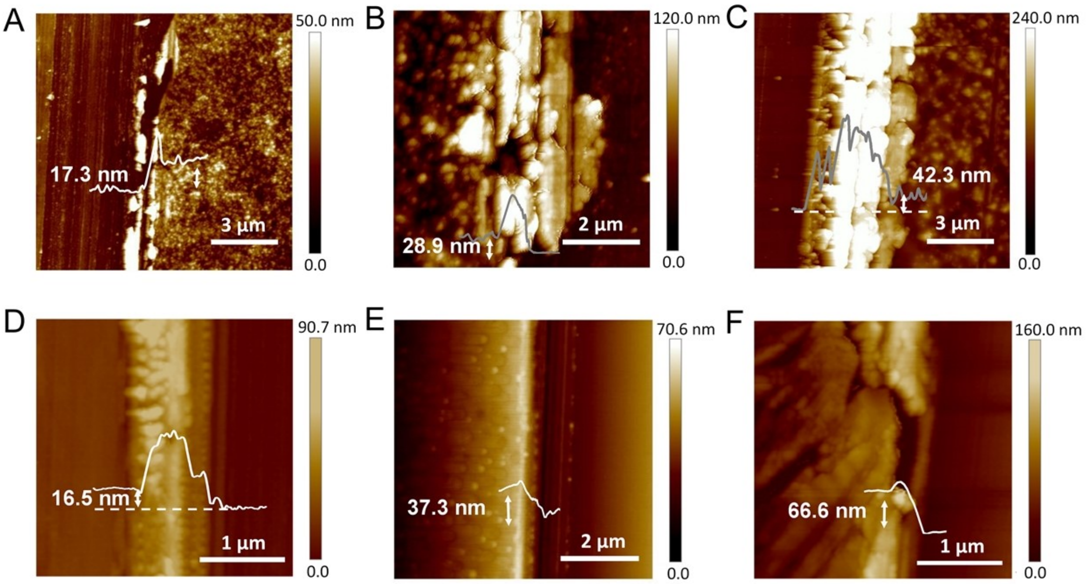

Figure S14. AFM images of TTF-NH_2 films on graphene: (A) after the first drop-casting (R_{RMS}:5.1 nm, 1.0 ×1.0 μm^2), (B) after the second drop-casting (R_{RMS}:10.3 nm, 1.0 ×1.0 μm^2), and (C) after the third drop-casting (R_{RMS}:11.8 nm, 1.0 ×1.0 μm^2); AFM images of spin-coated TTF-CHO films: (D) 3.2 mM solution at a spin rate of 600 rpm (R_{RMS}:1.2 nm, 1.0 ×1.0 μm^2), (E) 8.0 mM solution at a spin rate of 600 rpm (R_{RMS}:1.5 nm, 1.0 ×1.0 μm^2), and (F) 8.0 mM solution at a spin rate of 400 rpm (R_{RMS}:12.1 nm, 1.0 ×1.0 μm^2).

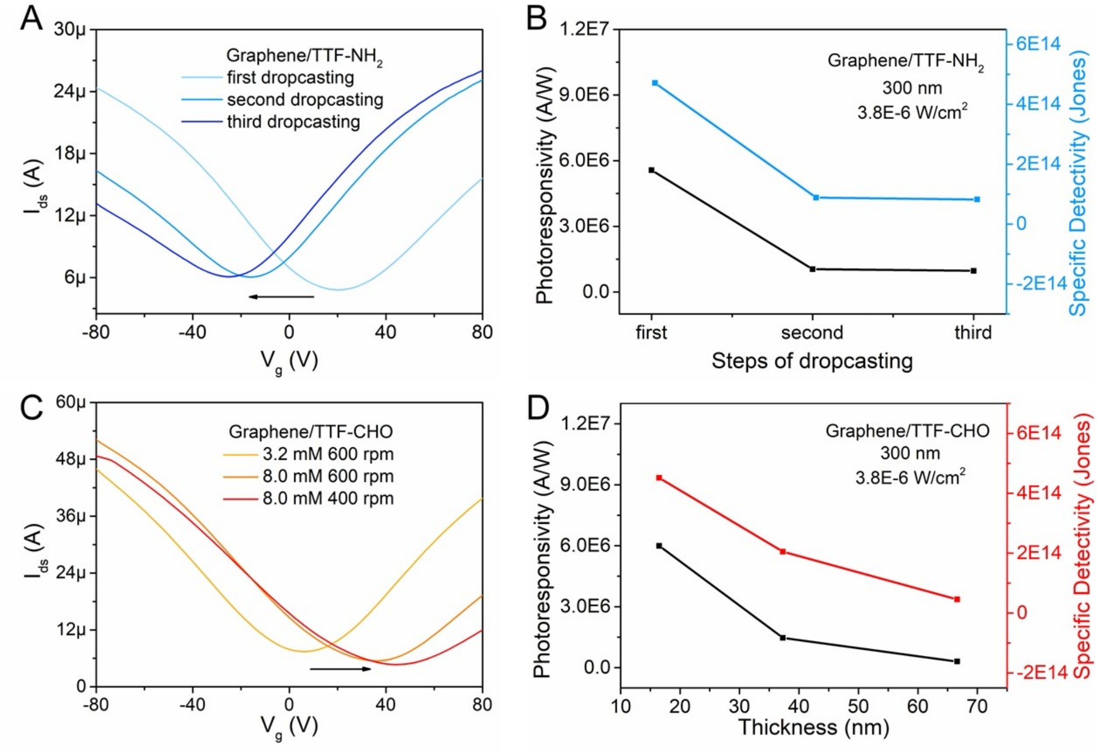

Figure S15. (A) Transfer curves of graphene/TTF-NH_2 hybrid device after step-by-step drop-casting process. (B) Photoresponsivity changes with thickness accumulation during step-by-step drop-casting. (C) Transfer curves and (D) Photoresponsivity changes of the graphene/TTF-CHO hybrid device at different concentrations and spin-coating speeds of the TTF-CHO solution.

Notes and references

1 G. Kresse and J. Hafner, *Phys. Rev. B*, 1993, **47**, 558-561.

2 G. Kresse and J. Furthmüller, *Phys. Rev. B*, 1996, **54**, 11169-11186.

3 P. E. Blöchl, *Phys. Rev. B*, 1994, **50**, 17953.

4 G. Kresse and D. Joubert, *Phys. Rev. B*, 1999, **59**, 1758-1775.

5 J. P. Perdew, K. Burke and M. Ernzerhof, *Phys. Rev. Lett.*, 1996, **77**, 3865-3868.

6 Gaussian 16, Revision A.03, M. J. Frisch, G. W. Trucks, H. B. Schlegel, G. E. Scuseria, M. A. Robb, J. R. Cheeseman, G. Scalmani, V. Barone, G. A. Petersson, H. Nakatsuji, X. Li, M. Caricato, A. V. Marenich, J. Bloino, B. G. Janesko, R. Gomperts, B. Mennucci, H. P. Hratchian, J. V. Ortiz, A. F. Izmaylov, J. L. Sonnenberg, D. Williams-Young, F. Ding, F. Lipparini, F. Egidi, J. Goings, B. Peng, A. Petrone, T. Henderson, D. Ranasinghe, V. G. Zakrzewski, J. Gao, N. Rega, G. Zheng, W. Liang, M. Hada, M. Ehara, K. Toyota, R. Fukuda, J. Hasegawa, M. Ishida, T. Nakajima, Y. Honda, O. Kitao, H. Nakai, T. Vreven, K. Throssell, J. A. Montgomery, Jr., J. E. Peralta, F. Ogliaro, M. J. Bearpark, J. J. Heyd, E. N. Brothers, K. N. Kudin, V. N. Staroverov, T. A. Keith, R. Kobayashi, J. Normand, K. Raghavachari, A. P. Rendell, J. C. Burant, S. S. Iyengar, J. Tomasi, M. Cossi, J. M. Millam, M. Klene, C. Adamo, R. Cammi, J. W. Ochterski, R. L. Martin, K. Morokuma, O. Farkas, J. B. Foresman, and D. J. Fox, *Gaussian, Inc.*, Wallingford CT, 2016.

7 Y. Lee, S. H. Yu, J. Jeon, H. Kim, J. Y. Lee, H. Kim, J.-H. Ahn, E. Hwang and J. H. Cho, *Carbon*, 2015, **88**, 165-172.

8 X. Liu, X. Luo, H. Nan, H. Guo, P. Wang, L. Zhang, M. Zhou, Z. Yang, Y. Shi and W. Hu, *Adv. Mater*, 2016, **28**, 5200-5205.

9 X. Liu, E. K. Lee and J. H. Oh, *Small*, 2014, **10**, 3700-3706.

10 Y. Xiong, Q. Liao, Z. Huang, X. Huang, C. Ke, H. Zhu, C. Dong, H. Wang, K. Xi, P. Zhan, F. Xu and Y. Lu, *Adv. Mater.*, 2020, **32**, 1907242.

11 X. Chen, X. Liu, B. Wu, H. Nan, H. Guo, Z. Ni, F. Wang, X. Wang, Y. Shi and X. Wang, *Nano Lett.*, 2017, **17**, 6391-6396.

12 Y. Yao, Q. Ou, K. Wang, H. Peng, F. Fang, Y. Shi, Y. Wang, D. I. Asperilla, Z. Shuai and P. Samorì, *Nat. Commun.*, 2021, **12**, 3667.

13 G. F. Jones, R. M. Pinto, A. De Sanctis, V. K. Nagareddy, C. D. Wright, H. Alves, M. F. Craciun and S. Russo, *Adv. Mater.*, 2017, **29**, 1702993.

14 K. P. Bera, G. Haider, M. Usman, P. K. Roy, H. I. Lin, Y. M. Liao, C. R. P. Inbaraj, Y. R. Liou, M. Kataria, K. L. Lu and Y. F. Chen, *Adv. Funct. Mater.*, 2018, **28**, 1804802.

15 M. A. Iqbal, A. Liaqat, S. Hussain, X. Wang, M. Tahir, Z. Urooj and L. Xie, *Adv. Mater.*, 2020, **32**, 2002628.

16 X. Liu, X. Chen, J. Yi, Z. Luo, H. Nan, H. Guo, Z. Ni, Y. Ding, S. Dai and X. Wang, *Org. Electron.*, 2019, **64**, 22-26.

17 G. Zhou, R. Sun, Y. Xiao, G. Abbas and Z. Peng, *Adv. Electron. Mater.*, 2021, **7**, 2000522.

18 G. Konstantatos, M. Badioli, L. Gaudreau, J. Osmond, M. Bernechea, F. P. G. De Arquer, F. Gatti and F. H. L. Koppens, *Nat. Nanotechnol.*, 2012, **7**, 363-368.

19 S. Qin, X. Qin, Q. Du, Y. Gan, Y. Zhang, A. Wang, X. Yan, R. Dong, Y. Liu, S. Li, C. Liu, W. Wang and F. Wang, *J. Mater. Chem. C*, 2022, **10**, 11710-11718.
